# Development of Cork Biocomposites Enriched with Chitosan Targeting Antibacterial and Antifouling Properties

**DOI:** 10.3390/molecules28030990

**Published:** 2023-01-18

**Authors:** Emanuel M. Fernandes, Flávia C. M. Lobo, Sara I. Faria, Luciana C. Gomes, Tiago H. Silva, Filipe J. M. Mergulhão, Rui L. Reis

**Affiliations:** 1 3B’s Research Group, I3Bs—Research Institute on Biomaterials, Biodegradables and Biomimetics, Headquarters of the European Institute of Excellence on Tissue Engineering and Regenerative Medicine, University of Minho, AvePark, Parque de Ciência e Tecnologia, Zona Industrial da Gandra, Barco, 4805-017 Guimarães, Portugal; 2 ICVS/3B’s—PT Government Associate Laboratory, 4806-909 Guimarães, Portugal; 3 LEPABE—Laboratory for Process Engineering, Environment, Biotechnology and Energy, Faculty of Engineering, University of Porto, Rua Dr. Roberto Frias, 4200-465 Porto, Portugal; 4 ALiCE—Associate Laboratory in Chemical Engineering, Faculty of Engineering, University of Porto, Rua Dr. Roberto Frias, 4200-465 Porto, Portugal

**Keywords:** polymer-matrix composites, particle reinforcement, extrusion, surface properties, green materials

## Abstract

The demand for bio-based and safer composite materials is increasing due to the growth of the industry, human population, and environmental concerns. In this framework, sustainable and safer cork-polymer composites (CPC), based on green low-density polyethylene (LDPE) were developed using melt-based technologies. Chitosan and polyethylene-graft-maleic anhydride (PE-g-MA) were employed to enhance the CPC’s properties. The morphology, wettability, mechanical, thermal, and antibacterial properties of the CPC against *Pseudomonas putida* (*P. putida*) and *Staphylococcus aureus* (*S. aureus*) were examined. The CPC showed improved stiffness when compared with that of the LDPE matrix, preferably when combined with chitosan and PE-g-MA (5 wt. %), reinforcing the stiffness (58.8%) and the strength (66.7%). Chitosan also increased the composite stiffness and strength, as well as reduced the surface hydrophilicity. The CPCs’ antibacterial activity revealed that cork significantly reduces the biofilm on the polymer matrix. The highest biofilm reduction was found with CPC containing cork and 5 wt. % chitosan for both *P. putida* (54% reduction) and *S. aureus* (36% reduction), confirming their potential to extend the lifespan of products for packaging and healthcare, among other applications. This work leads to the understanding of the factors that influence biofilm formation in cork composites and provides a strategy to reinforce their behavior using chitosan.

## 1. Introduction

In times of high interest for materials with a dominant content of renewable resources, lignocellulosic materials present a highly attractive feedstock. Along with this, materials are also predicted to show effective antimicrobial and antifouling properties to cope with current safety demands. The development of new materials avoiding adverse health risks and circular alternatives for preserving resources for future generations is required [1,2,3]. In this light, the design and development of sustainable solutions comprising antibacterial and/or antifouling properties will address those problems. Biocomposites containing lignocellulosic fractions combined with biopolymers from natural sources would be an ideal solution to obtain sustainable materials. Lignocellulosic materials represent a renewable, biodegradable, lightweight, and abundant source, making them attractive for the development of sustainable products [4,5,6,7]. Among other renewable alternatives, cork-polymer biocomposites (CPCs) have attracted the attention of scientists and companies [8,9,10]. Cork is obtained from the outer bark of the oak tree (*Quercus suber* L.), which can be harvested every 9 to 12 years without harming the tree [11]. Chemically, cork is very different from other plant tissues (such as wood), containing on average 43% suberin, followed by 22% lignin, 19% polysaccharides (cellulose and hemicelluloses), and 16% extractives [12]. Cork presents an anisotropic closed cellular structure, as it is a lightweight material with good elasticity and is non-toxic, with low permeability to liquids and gases, acting as a good thermal, acoustic, and electrical insulator, and exhibiting a near-zero Poisson coefficient [11,13,14]. Recently, a study using pure cork showed that it presents high antibacterial activity against the Gram-positive bacterium *Staphylococcus aureus*, enabling a bacterial reduction close to 100%, whereas for the Gram-negative bacterium *Escherichia coli*, the maximum bacterial reduction was 36% [15].

On the polymer market side, polyolefins are the commonly used plastics in our daily life [3,16], but they do not possess antibacterial properties [17]. Cork has been combined with polyolefins such as polypropylene (PP) [10,18,19], polyethylene (PE) [10,20], and bio-based PE (Bio-PE) [9] through melt-based technologies. Nevertheless, there is no information on cork behavior in polymer-matrix composites in terms of antimicrobial activity or antifouling properties. Focusing on bio-renewable materials, in this work, granulated cork was compounded via twin-screw extrusion with a bio-based low-density polyethylene (LDPE), with the aim to improve its antibacterial activity and to protect the surfaces from biofilm growth. The worldwide capacity of bio-based plastics is increasing; thus, we selected a bio-based LDPE as a green matrix for the development of biocomposites with cork. The first company to produce the Bio-PE, synthetized using ethanol from sugarcane, was Braskem (a Brazilian company). Braskem commercializes polymers for automotive, packaging, personal care, and toys sectors [21], making the Bio-PE equally processable and recyclable [3].

During the lifetime of materials, microorganisms may cause illness in humans via water, food, or other vectors. Bacteria and microorganisms possess the natural tendency to attach to solid surfaces, and then multiply and surround themselves with a slimy matrix of extracellular polymeric substances (EPS), forming a biofilm [7,22,23]. The formation and development of a biofilm depends on several factors, such as the bacterial strain(s), the material surface properties, and the present environmental conditions (pH, temperature, and nutrients) [24]. Biofilms are the main things responsible for biofouling (the accumulation of microorganisms in surfaces), biocorrosion, and reservoir souring, causing several problems for different industries [25,26]. Antifouling and antimicrobial surfaces are extensively studied for biofilm control. Antifouling surfaces are resistant to the adsorption of contaminants, whereas antimicrobial surfaces kill or inhibit microbial growth [27]. Some studies reveal the use of polymers showing antibacterial properties (such as chitosan (CHT)) or polymer matrices with the inclusion of metallic nanoparticles, zinc oxide, or silver nanoparticles [28,29]. Generally, chitosan is obtained through the alkaline deacetylation of chitin, and it is composed of 70–90% of d-glucosamine and 10–30% of *N*-acetyl-d-glucosamine units linked by β (1→4) glycosidic connection. Among the antimicrobial natural biopolymers, chitosan was chosen to improve the behavior of CPC materials, since it presents remarkable characteristics, being non-toxic, biodegradable, biocompatible, and most importantly, having antibacterial activity [29,30,31]. Therefore, this study intends to evaluate the behavior of cork as an antibacterial and antifouling agent in polymer-matrix biocomposites and to improve those properties by reinforcing it with chitosan to ensure the development of safer and more sustainable composite materials. This is the first study addressing the effect of cork or its combination with chitosan in polymer-matrix biocomposites obtained via melt-based technologies against bacterial biofilm formation.

## 2. Results and Discussion

### 2.1. Density and Mechanical Properties

**Figure 1:**
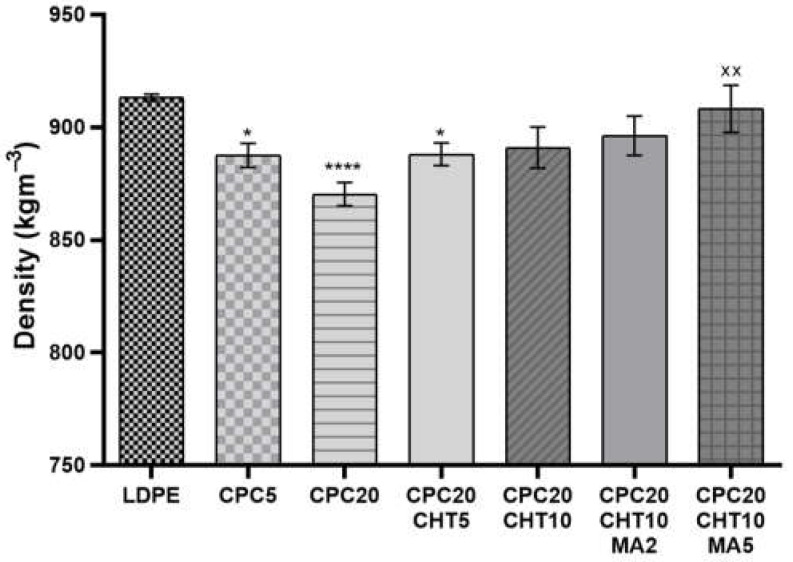
The density of the pristine low-density polyethylene (LDPE) and its cork biocomposites (CPC) containing chitosan (CHT) with or without maleic anhydride (MA). The significance levels between groups were 1 symbol—*p* < 0.05, 2 symbols—*p* < 0.01, and 4 symbols—*p* < 0.0001 related to: * LDPE; x CPC20.

The density of each developed CPC is reported in Figure 1. The density of the pristine bio-based LDPE used as a matrix on the biocomposites is 919.0 kg/m^3^ and, by adding granulated cork, the density was reduced by 2.8% for 5 wt. % of cork and 4.7% for the composition with 20 wt. % of cork. This decrease in the biocomposite weight with the increase of cork content was previously observed in polymer biocomposites containing powder or granulated cork [18]. For CPC formulations containing 20 wt. % of cork, it was noticed that the inclusion of chitosan significantly increased the biocomposite density.

A medium molecular weight chitosan was selected for this study, and the incorporation of 10 wt. % of chitosan resulted in an increase of density of 2.4% compared with the CPC20. The use of the coupling agent polyethylene-graft-maleic anhydride (PE-g-MA) during the compounding via extrusion and followed by compression molding also promoted an additional increase of the biocomposite density to the approximate values of the LDPE matrix.

**Figure 2:**
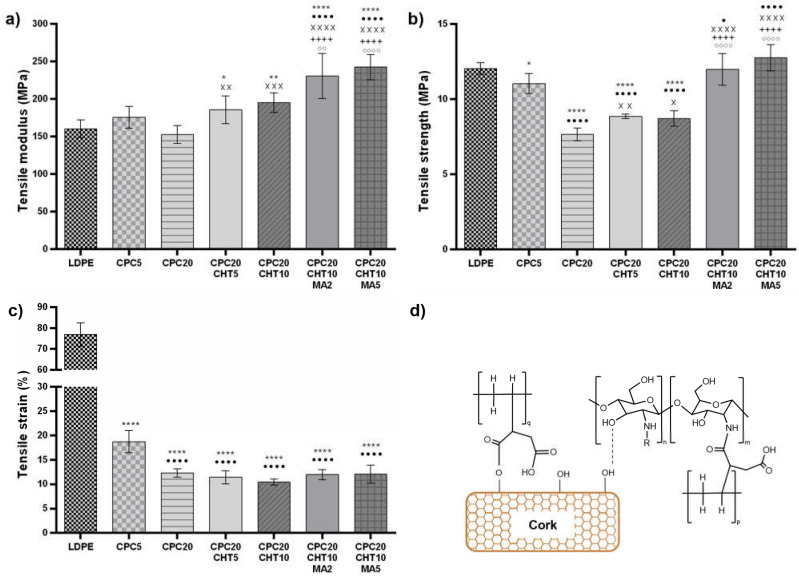
The mechanical properties of the pristine LDPE and the CPC with and without chitosan and the coupling agent PE-g-MA, for the tensile (**a**) elastic modulus, (**b**) maximum strength, and (**c**) strain at maximum load. The significance levels between groups were 1 symbol—*p* < 0.05, 2 symbols—*p* < 0.01, 3 symbols—*p* < 0.001 and 4 symbols—*p* < 0.0001 related to: * LDPE; • CPC5. x CPC20, + CPC20CHT5, ○ CPC20CHT10; (**d**) scheme of the bounds established between the main components present in the biocomposite.

Considering previous works, the mechanical properties of CPC materials are mainly dependent on the (i) type of matrix selected; (ii) cork content; (iii) cork–matrix interface compatibility, and (iv) applied melt-based technology [8,10,19,32,33,34]. In this work, uniaxial tests under tensile load were applied to assess the mechanical performance of the developed green materials and understand the contribution of each constituent in the biocomposite. Figure 2 and Table S1 show the variations of the elastic modulus, the maximum strength, and the strain at the maximum load of the bio-based LDPE matrix and its CPC. As expected, the addition of granulated cork to the LDPE led to a decrease in the biocomposite’s tensile strength. However, since the LDPE shows an elastic modulus of 165.35 MPa, it was possible to reinforce the matrix stiffness by adding 5 wt. % of cork, corresponding to a stiffness increase of 6.3%. As expected, increasing the cork content leads to a reduction in the biocomposite stiffness and decreases the tensile strain of those systems. The low mechanical properties of cork particles under tensile load and the chemical nature of the constituents (as cork is more polar compared to the hydrophobic nature of the bio-based matrix) explains this effect and it is in agreement with previous works [10,18]. Conversely, the increase of natural components based on chitosan in 5 wt. % or 10 wt. % to the CPC20 induces an increase in the CPC stiffness and a small increase in the tensile strength. This unexpected increment of the tensile strength in this hybrid system containing cork and chitosan might result from the flake shape of chitosan [35], which is effective in transferring the stress from the LDPE matrix to the filler. Moreover, during the twin-screw extrusion process, the constituents based on LDPE combined with cork and chitosan are under shear stress during melt mixing, which promotes their physical interaction.

Thus, the hydroxyl groups of the cork surface particles and the hydroxyl and amino groups present in chitosan can react, forming intermolecular hydrogen bonds (Figure 2d). In this way, they also contribute to the observed reinforcement of the stiffness and the maximum tensile strength properties of the CPC materials. The interaction between chitosan and other polymer-matrix biocomposites with natural fibers has been previously reported [36,37]; however, the mechanical reinforcement does not always occur. Biocomposites based on polylactic acid (PLA) and wood flour have been compounded with chitosan, assuming that the chitosan addition at the interface between PLA and wood has formed hydrogen bonds to the carbonyl groups of PLA. When combined with 10 wt. % of chitosan, no significant differences in the mechanical behavior of the biocomposites were observed, being the mechanical properties dependent on the wood flour content [36]. In other works, by combining polyvinyl chloride (PVC) with wood flour, the presence of chitosan increased the flexural strength and the flexural modulus [37] and reduced the fusion torque during processing of those biocomposites [38], due to the promoted interfacial adhesion between the polymer matrix and the wood flour. The increase in the mechanical parameters was attributed to the acid-base interaction between the PVC and chitosan, showing that this natural polymer enhances the properties of PVC/wood flour biocomposites [37,38]. Furthermore, in this study, the used PE-g-MA also contributed to an effective increase in the tensile properties of the CPC materials. For a higher concentration of PE-g-MA (5 wt. %) and compared with the CPC20, a significant reinforcement of the elastic modulus (an increase of 58.8%) and the tensile strength (an increase of 66.7%) was obtained without compromising the strain at maximum load. This mechanical reinforcement on the CPC properties obtained using PE-g-MA also corresponds to a significant reinforcement of the stiffness of the neat bio-based LDPE in 46.7%. The increase in the compatibility between the hydroxyl groups at the cork surface and the anhydride part contributes to a reduction in the interfacial tension, increasing the interfacial adhesion between the cork and the LDPE matrix [39] (Figure 2d). Moreover, previous studies on the processing and rheological behavior of CPC and a similar coupling agent (i.e., maleic anhydride) [19,33] showed that the use of the coupling agent (PE-g-MA) contributes to a decrease in CPC viscosity. By reducing the viscosity, the coupling agent also promotes a plasticizing effect that will contribute to disperse the cork and chitosan particles in the matrix during the melt-based process. In addition, it is proposed that chitosan can also establish chemical bounding with PE-g-MA. As indicated in Figure 2d, the maleic anhydride acts as a cross-linking agent between the two biopolymers (chitosan and polyethylene) [40] and cork-polyethylene. These established covalent bounds and the hydrogen bounds established between cork and chitosan support the increase of the tensile modulus and tensile strength.

### 2.2. Thermal Properties

**Figure 3:**
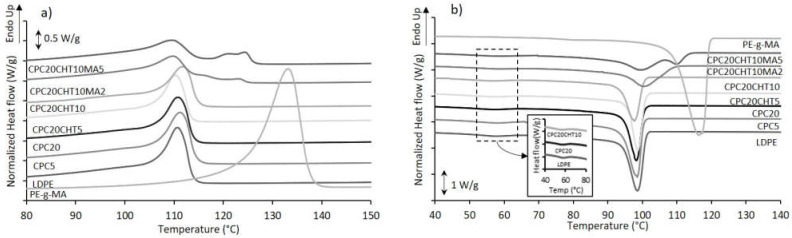
The representative DSC thermograms of CPC materials using a bio-based LDPE as a matrix on cork biocomposites: (**a**) second heating step and (**b**) first cooling step.

The thermal behavior of all prepared materials was investigated by differential scanning calorimetry (DSC). Representative DSC curves for the biocomposites based on the bio-based LDPE are shown in Figure 3, and the main thermal events are reported in Table 1.

The crystallization temperature (T_c_) of the pristine LDPE occurs at 100.3 °C; however, in the thermogram and in the insert image of Figure 3b, a small peak is shown at 60 °C in the crystallization curve. Wang et al. [41] proposed that, during the cooling process, it is possible that a small quantity of highly branched LDPE cannot crystallize with the major component at 100.3 °C. This event is also present in all of the CPCs’ thermograms. In the CPC5 and the CPC20, the addition of cork promoted a slight increase in this T_c_. However, those results are not significantly different and, for that reason, it was not possible to observe the nucleation effect of cork particles described in the literature [18,42,43]. Moreover, CPC materials containing chitosan do not show an increase in the melting temperature (T_m_); this can be justified by the poor miscibility of both polymers. The LDPE matrix is hydrophobic and repels the hydrophilic chitosan structure, producing an immiscible blend [44,45].

The CPCs that were produced with a coupling agent, CPC20CHT10MA2, and CPC20CHT10MA5, present three peaks in the thermograms (Figure 3a). This effect is reported in the literature for blends based on LDPE and high-density polyethylene (HDPE). Since the PE-g-MA coupling agent used is mainly based on HDPE, the corresponding melting temperature was observed. Munaro et al. [46] proposed that these three peaks refer to three average-size crystallites formed by the LDPE and the HDPE separately and/or through co-crystallization. The thermal event at around 100 °C refers to the LDPE matrix’s melting temperature, and the second event, around 120 °C, belongs to the melting of PE-g-MA crystals. Considering that the coupling agent is HDPE based, the melting temperature is higher than that of the LDPE matrix. The intermediary peak could also indicate that crystals containing LDPE and HDPE segments were formed [47].

### 2.3. Contact Angle

**Figure 4:**
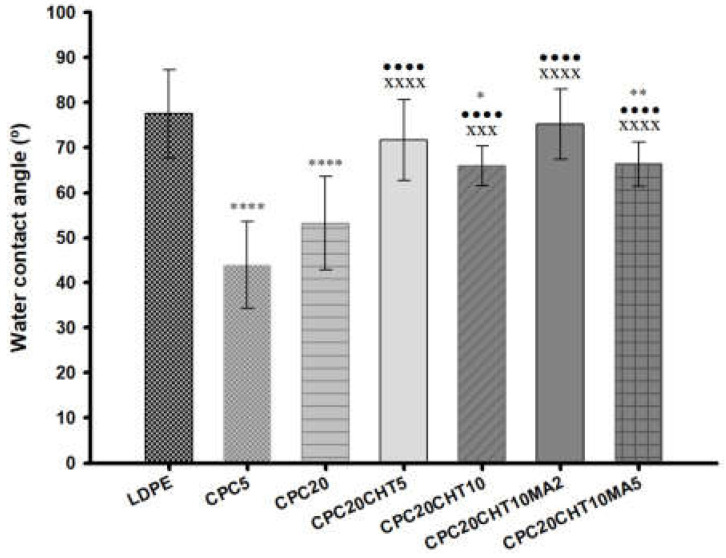
The water contact angles of the pristine LDPE and the CPC with and without chitosan and a coupling agent. The means ± standard deviation (SD) are illustrated. The significance levels between groups were 1 symbol—*p* < 0.05, 2 symbols—*p* < 0.01, 3 symbols—*p* < 0.001 and 4 symbols—*p* < 0.0001 related to: * LDPE; • CPC5. x CPC20, + CPC20CHT5, ○ CPC20CHT10, ■ CPC20CHT10MA2.

All produced materials were characterized by water contact angle measurements (Figure 4) to evaluate the surface wettability properties. Typically, a surface is hydrophilic when its static water contact angle (θ) is lower than 90° and is hydrophobic when θ is higher than 90°. LDPE is commonly used as packaging material since it has relatively good transparency, flexibility, and moisture resistance.

The measured contact angle of pristine LDPE was approximately 80°, which is in agreement with the hydrophobicity described for this polymer [26]. With the addition of 5 wt. % (CPC5) and 20 wt. % (CPC20) of cork, the water contact angle decreased to 44° and 53°, respectively, which indicates that the addition of granulated cork in the polymeric matrix decreased the hydrophobicity of the biocomposite surface after processing. Among the unique properties of cork, its impermeability to liquids is often mentioned, showing a contact angle of around 90° at 24 °C [48]. However, cork can also be considered to show an intermediate hydrophobic/hydrophilic behavior [49]. Cork is a cellular tissue without intercellular openings or communication structures at the micrometer level, such as those present in wood; however, it has been suggested that water can diffuse through cell walls [48]. In addition, the water at the cork surface can sorb on hydrophilic sites that are constituted by hydroxyl and methoxyl groups through hydrogen bonds, and then sorption continues via water cluster formation all over the hydrophilic sites [49]. This supports the decrease of the water contact angle obtained in the cork biocomposites; however, there was no visual evidence of substrate swelling. When chitosan was added to the biocomposites (CPC20CHT5 and CPC20CHT10), the obtained water contact angles for these two materials were 72° and 66°, respectively. Those values were still slightly lower than those of pristine LDPE, which might be related to the hydrophilic character of chitosan [44,45]. Moreover, when compared with the biocomposites containing only cork, a small increase in the surface hydrophobicity was noticed. This might be related to the surface topography, where the material roughness becomes smoother with the chitosan, increasing the water contact angle as observed in the scanning electron microscopy (SEM) micrographs. In addition, the surface roughness also results from the detachment of the Teflon sheet from the biocomposite surface after the compression molding process. The literature indicates that the degree of roughness and surface chemistry affects the wettability of a solid material [50,51], and it has been described that with the increase in surface roughness, the apparent contact angle decreases for hydrophilic materials [50]. Finally, the addition of 2 wt. % (CPC20CHT10MA2) or 5 wt. % (CPC20CHT10MA5) of a coupling agent (PE-g-MA) to the CPC with the highest amount of chitosan did not significantly change the surface wettability. However, for a low concentration of coupling agent and higher chitosan content (CPC20CHT10MA2), a slight increase in the contact angle was observed. This is probably due to the hydrophobic nature of the coupling agent that improved the dispersion of the constituents in the matrix.

### 2.4. Water Absorption

**Figure 5:**
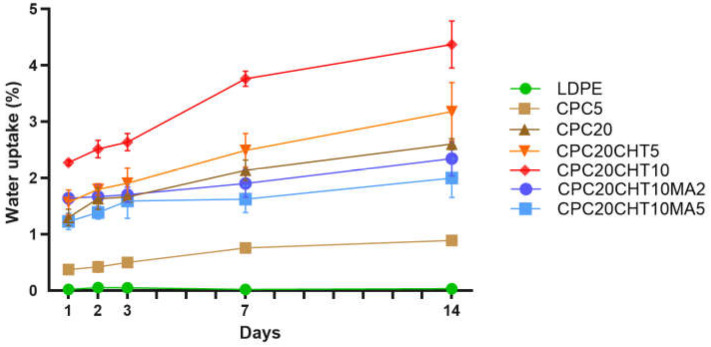
The water uptake behavior of the pristine LDPE and its cork biocomposites.

The water absorption behavior of the pristine LDPE and the different CPC was examined as a function of time for 14 days, as shown in Figure 5. Polyolefins do not present chemical bonds that are easily hydrolysable, and they almost absorb no water. Thus, the bulk properties are not affected by long periods of water immersion. The water uptake in the pristine LDPE was negligible after 14 days of immersion, resulting in a value of (0.03 ± 0.01)%. Typically, LDPE is known as one of the most common packaging materials since it has relatively good transparency, flexibility, and resistance to moisture [39]. One promising feature of polymer-matrix biocomposites with cork is their low water absorption. The addition of 5 wt. % (CPC5) and 20 wt. % (CPC20) of cork resulted in a reduced water uptake of (0.76 ± 0.04)% and (2.13 ± 0.18)%, respectively, after 7 days of immersion. After 14 days, those values slightly increased to (0.9 ± 0.8)% for CPC5 and (2.60 ± 0.10)% for CPC20. Cork is more hydrophobic than other natural fibers. However, it also absorbs moisture. The water at the cork surface can sorb on hydrophilic sites constituted by hydroxyl and methoxyl groups through hydrogen bonds, and then sorption continues through water cluster formation around the hydrophilic sites [40,41]. Thus, the increase in cork content increased the water uptake of the biocomposites when compared with the pristine LDPE.

This behavior, as well as the ability of cork to promote dimensional stability and low values of water uptake when compared with natural fiber biocomposites, is in agreement with previous works using melt-based technologies to produce CPC and hybrid CPCs (i.e., cork with natural fibers) [9,17,18]. As expected, with the addition of chitosan particles, an increase in the water uptake due to chitosan’s hydrophilic nature was observed. The chitosan structure possesses several hydroxyl and amino groups that can establish hydrogen bonds with the water molecules, as well as the hydroxyl and aldehyde groups at the end of the polymeric chain [40]. On the other hand, the reactions between PE-g-MA with cork or chitosan (Figure 2d) contributed to the observed decrease in the water absorption of the biocomposites. The PE-g-MA can establish a covalent bond with both cork and chitosan, decreasing the availability of sites used to establish hydrogen bonds [18,40].

### 2.5. Visual Aspect and Morphology

**Figure 6:**
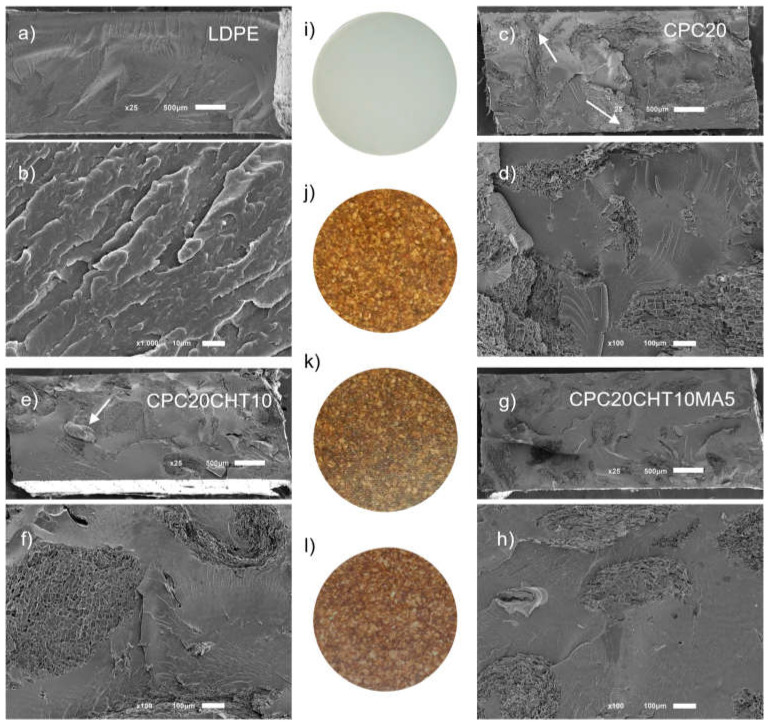
Cross-section morphology for the bio-based LDPE (**a**,**b**), CPC20 (**c**,**d**), CPC20CHT10 (**e**,**f**), and CPC20CHT10MA5 (**g**,**h**) and the visual aspect of the developed materials for the antimicrobial tests, with 35 mm of diameter and based on LDPE (**i**); CPC20 (**j**), CPC20CHT10 (**k**) and CPC20CHT10MA5 (**l**). (**a**,**c**,**e**,**g**) Magnification at ×25 (scale bar 500 µm) and (**b**,**d**,**f**,**h**) higher magnification of ×100 (scale bar 100 µm).

The appearance of the produced materials for the antibacterial tests (35 mm of diameter) and the cross-section morphology are presented in Figure 6. The typical cryogenic fracture of the polymer matrix is shown in Figure 6a,b, while with 20 wt. % of granulated cork, the distribution of cork in the matrix was observed (Figure 6c). In addition, this figure shows arrows indicating that, after the processing, some of the cork particles seemed to be in contact with the surface. At higher magnification, as presented in Figure 6d, a good physical adhesion was observed between the main composite constituents, the cork, and the LDPE matrix. In the micrograph of Figure 6e indicated by the arrow, the flake shape of a chitosan particle was observed in the condition containing 10 wt. %. Since chitosan is an immiscible component and does not melt, it was possible to observe the particle; however, this result was not clear in other conditions containing chitosan. By increasing the complexity of this system with the addition of a coupling agent based on PE-g-MA, no relevant changes were observed as compared to the previous conditions, where the good interaction of the cork polymer with the chitosan particles was maintained.

As reported in previous studies [18,42], the coupling agent improves the interfacial properties of the polymer filler by reducing the interfacial tension and, simultaneously, the agglomeration tendency of filler particles. Typically, the coupling agent reacts with the filler surface and at least one side group reacts or stays compatible with the polar functional groups of the g-MA polymer. Considering the cork surface, the available hydroxyl groups can react with the coupling agent, making the final material more hydrophobic (Figure 4), and improving its mechanical properties (Figure 2).

In terms of visual aspect, the developed biocomposite materials show a good fill of the LDPE matrix by using 20 wt. % of granulated cork. The addition of chitosan or its use in combination with the coupling agent, the brown color of the biocomposites tends to intensify, as shown in Figure 6, images j, k, and l, respectively.

**Figure 7:**
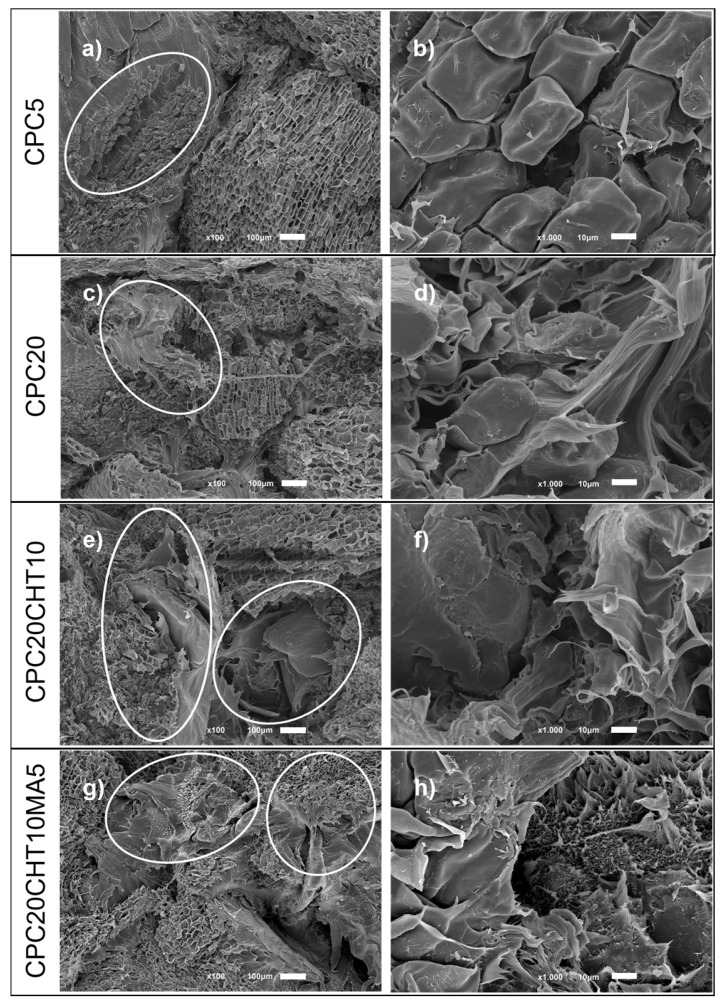
SEM analysis of damage and fracture mode of the biocomposites after tensile tests: (**a**,**b**) cork pull-out and interface debonding; (**c**,**d**) matrix deformation and interface debonding; (**e**,**f**) matrix cracking and cork breakage; (**g**,**h**) matrix cracking and cork breakage. Magnification at ×100 (**a**,**c**,**e**,**g**) and higher magnification of ×1000 (**b**,**d**,**f**,**h**).

The fracture morphology of the CPC materials after the uniaxial tensile tests was assessed using SEM, as shown in Figure 7. In the micrographs, the differences between the surface roughness features are rather significant. For biocomposites containing only cork, the damage fracture occurs at the polymer cork interface, where the debonding of cork is marked (Figure 7a,c). At higher magnification (Figure 7b), the pattern of non-radial direction of cork cellular structure observed to be imprinted on the polyethylene-matrix resulted from the cork pull-out. The cork debonding confirms that cork acts as a natural filler and contributes to the decrease in the mechanical performance (Figure 2). Considering the field of natural fibers composites and the previous studies that reveals poor fiber-matrix adhesion, they report that the dominant deformation mechanism is debonding especially when it occurs in large particles and high strain rates [52,53]. The morphology also reveals the stretching of the bio-based LDPE matrix (Figure 7d), indicating a ductile fracture behavior. For biocomposites containing chitosan (Figure 7e,f) and, preferably, chitosan with a coupling agent (Figure 7g,h), the fracture occurs in plane with the surface with reduced stretching of the LDPE matrix and a clear improved cork-matrix interface. 

By using a coupling agent, the stretching of the polyethylene matrix is almost unobservable, causing a brittle fracture to occur. This result is in agreement with the increase in the stiffness and strength obtained under tensile load. The observed improvement in the cork–polymer interfacial adhesion is also in agreement with the microstructure of previous works containing a similar coupling agent with maleic anhydride in composites with higher cork content [42] and in hybrid cork composite materials that are reinforced with natural fibers [32,54]. Deformation mechanisms can be also distinguished on SEM micrographs, indicating that they occur during the deformation process.

### 2.6. Antibacterial Activity

**Figure 8:**
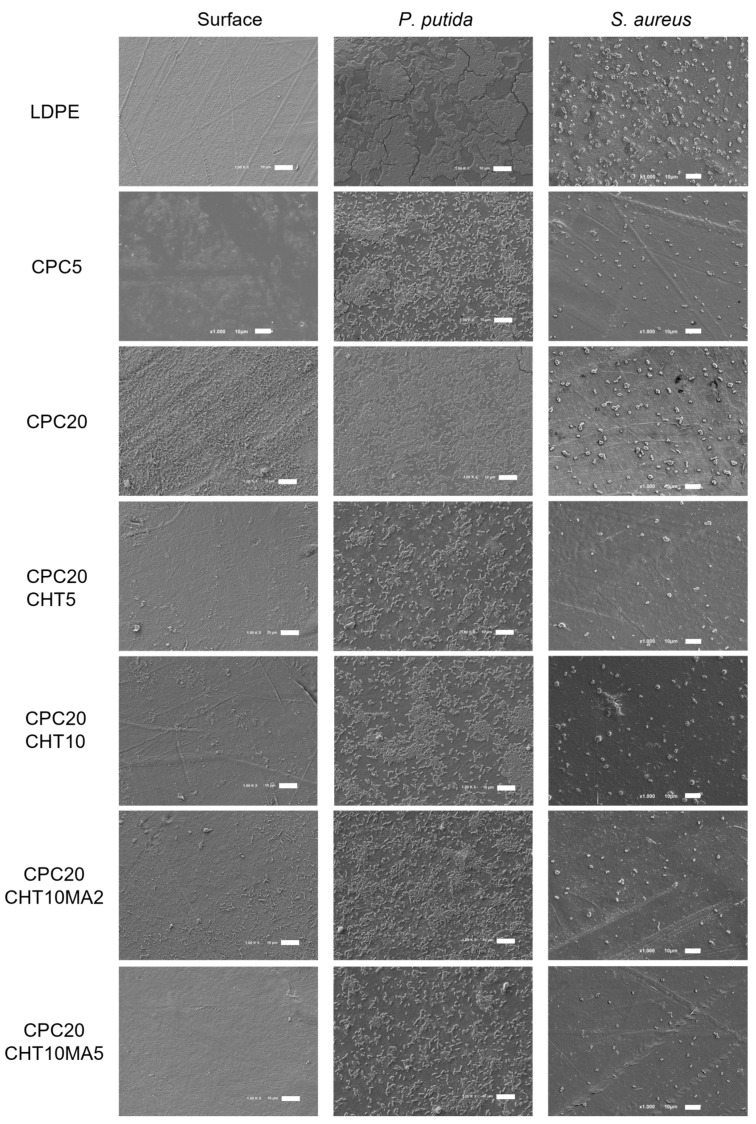
SEM images of neat LDPE and biocomposites (left column), and of *P. putida* (middle column) and *S. aureus* biofilms (right column) formed after 3 days of incubation. The scale bar represents 10 µm, magnification of ×1000.

**Figure 9:**
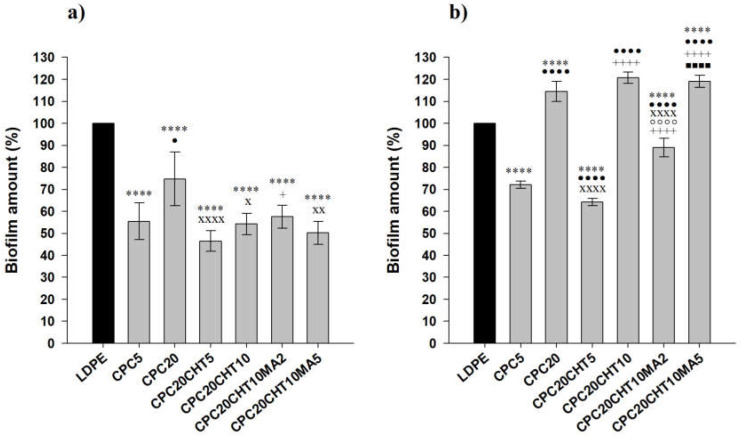
*P. putida* (**a**) and *S. aureus* (**b**) biofilm amount on pristine LDPE and biocomposites. The normalized mean values ± SD for three independent experiments with two technical replicates are each illustrated. The significance levels between the groups were 1 symbol—*p* < 0.05, 2 symbols—*p* < 0.01, 3 and 4 symbols—*p* < 0.0001 related to: * LDPE; • CPC5. x CPC20, + CPC20CHT5, ○ CPC20CHT10, ■ CPC20CHT10MA2.

The antibacterial activity of LDPE and the produced biocomposites were evaluated in conditions mimicking the storage conditions of packaged food products, namely a short incubation period (3 days), a refrigeration temperature of 5 °C, and static conditions. Furthermore, two different biofilm-forming bacteria typically associated with the food environment were used: *Pseudomonas putida* (*P. putida*) (a Gram-negative bacterium isolated from the salad processing industry) and *Staphylococcus aureus* (*S. aureus*) (a model Gram-positive bacterium). The spatial distribution of the biofilms formed by both bacterial strains on the surfaces was analyzed via SEM (Figure 8), whereas the biofilm amount was quantified by the crystal violet (CV) staining method (Figure 9).

Looking at the SEM micrographs (Figure 8), it is evident that *P. putida* formed a higher amount of biofilm than *S. aureus* on all tested surfaces. While the *Pseudomonas* strain originated large cell clusters that were homogeneously distributed and covered almost the entire available surface area, *S. aureus* adhered to the surfaces, forming very small aggregates comprising approximately five cells at the end of 3 days. 

Furthermore, the produced biocomposites were all effective in reducing biofilm formation by *P. putida* when compared to LDPE (a 44% reduction on average, Figure 9a), contrary to what was observed for *S. aureus*, where three out of six materials showed an increased biofilm amount compared to the control polymeric matrix (Figure 9b). For this Gram-positive strain, the three biocomposite materials shown to have antibacterial activity (CPC with 5 wt. % cork, CPC with 20 wt. % cork and 5 wt. % chitosan, and CPC with 20 wt. % cork, 10 wt. % chitosan and 2 wt. % PE-g-MA) only reduced 25% of the biofilm amount on average when compared to pristine LDPE (Figure 9b).

Regardless of the tested strain, CPC containing 5 wt. % cork was more effective in reducing biofilm formation than the biocomposite with 20 wt. % cork (Figure 8 and Figure 9). This could be related to the existence of some microfilaments of the polymer at the surface of CPC with 20 wt. % cork, as observed using SEM (Figure 8c,d). These artifacts on the surface were probably caused by the production process itself, namely by the compression molding and the removal of the plate from the Teflon sheet, leading to an increase in adhesion sites for bacteria. Additionally, irregularities on the surface of CPC with 20 wt. % of cork may be associated with the presence of cork granules on the top of the surface, as shown in Figure 7, which promoted the biofilm growth as opposed to the smoother surface of CPC containing 5 wt. % cork (Figure 8). 

To increase the antibacterial effect of cork-based biocomposites, the incorporation of chitosan particles at a low concentration (5 wt. %) was tested. As chitosan has a high commercial value, we bet on the potential of cork at higher concentrations (20% instead of 5 wt. %) because it is an easily obtainable industrial residue and thus has a lower commercial value. The CPC20 with 5 wt. % chitosan in the composition showed to be more effective in reducing both *P. putida* and *S. aureus* attachment than CPC20 without chitosan. In fact, there was a 36% decrease in the amount of *P. putida* biofilm (Figure 9a) and a 54% decrease in the *S. aureus* biomass (Figure 9b) when chitosan was integrated into the LDPE–cork system. Nevertheless, increasing chitosan concentration from 5 to 10 wt. % did not have the intended antibiofilm effect on both *P. putida* and *S. aureus* biofilms; while the amount of *P. putida* biofilm was similar to that of the CPC20 with 5 wt. % chitosan (Figure 9a), there was an increase in biofilm formation for *S. aureus* (Figure 9b). Previous works have demonstrated that chitosan concentration can affect antimicrobial activity [55,56]. Chitosan was shown to bind to the negatively charged cell surface of Gram-positive and Gram-negative organisms, disturbing the bacterial cell membrane and inducing leakage in intracellular constituents [57] when present in lower concentrations. This bactericidal effect can explain the delay in the biofilm development on biocomposite surfaces with only 5 wt. % chitosan. Contrarily, the protonated chitosan may coat the cell surface and prevent leakage [57] at higher concentrations, thus enabling the multiplication of biofilm cells. As discussed before, the coupling agent (based on maleic anhydride) was added to promote the mechanical properties of the biocomposites, particularly when higher amounts of cork and chitosan were used.

On the contrary, the protonated chitosan may coat the cell surface and prevent leakage [57] at higher concentrations, thus enabling the multiplication of biofilm cells. As pointed out before, the coupling agent (based on maleic anhydride) was added to promote the mechanical properties of the biocomposites, particularly when higher amounts of cork and chitosan were used. As expected, the results show that the coupling agent at 2 or 5 wt. % had no significant effect on the reduction of biofilm formation by both strains (Figure 9), except for when *S. aureus* was exposed to CPC20CHT10 with 2 wt. % maleic anhydride, in which case a decrease of about 30% in biofilm development was detected compared to CPC20CHT10 without a coupling agent (Figure 9b). 

Overall, this work shows that cork in a low concentration was effective to reduce the biofilm of neat LDPE. In addition, a CPC combining 20 wt.% cork and 5 wt. % chitosan was the material with the best antibiofilm performance against both *P. putida* (54% biomass reduction) and *S. aureus* (36% biomass reduction).

## 3. Materials and Methods

### 3.1. Materials

The granulated cork with a density of 58 kg/m^3^ and an average particle size of 0.5 to 1 mm was supplied by Cork Supply Portugal. The polymeric matrix selected was a green low-density polyethylene (LDPE SPB208) made from a renewable source, the ethanol sugarcane and supplied from Braskem (São Paulo, Brazil), displaying a melt-flow index (MFI) of 22 g/10 min (190 °C/2.16 kg), according to the standard of American Society for Testing and Materials (ASTM) D1238, with a melting temperature of 104 °C and a minimum 95% renewable carbon content. A coupling agent based on polyethylene grafted with maleic anhydride (PE-g-MA), comprising 0.5–10 wt. % of MA (Exxelor PE 1040), displaying an MFI of 1.4 g/10 min (190 °C/2.16 kg), and melting temperature of 131.3 °C, was supplied by ExxonMobil, Hanover, Germany. Chitosan (CHT) obtained from crab shells (practical grade), presenting a molecular weight of around 190–310 kDa, was purchased from Sigma-Aldrich, Taufkirchen, Germany.

### 3.2. Biocomposites Manufacturing

Prior to the extrusion process, the polymeric matrix was reduced to a granule size lower than 0.5 mm in an ultra centrifugal mill from Retsch (Haan, Germany). The LDPE and cork were dried at 60 °C overnight to remove the excess moisture in these materials. The compositions of the CPC and the compounding conditions are presented in Table 2. All of the CPC materials that are designated by CPC20 represent a ratio in terms of LDPE/Cork of 80/20 wt. %. For processing the materials, a co-rotating twin-screw extruder system from Rondol SCF (Nancy, France) was used, with screws of 16 mm diameter, a length to diameter ratio (L/D) of 25, and a single strand die of 3 mm. The compounding conditions are presented in Table 2.

The CPC filament was cooled in a water bath and cut using a pelletizer SCHEER (Stuttgart, Germany) and dried at 60 °C for 24 h. The CPC pellets were further compressed using a hot press (Carver, USA) to produce plates with 2 mm of thickness. The molding temperature was set at 145 °C and the pellets were kept for 6 min without applying pressure, followed by 3 min under pressure of 4 tons. Moreover, the water cooling period of the plates was performed under pressure. Then, tensile bars with a neck cross-section area of 2 mm × 4 mm for mechanical tests and discs with a diameter of 35 mm for the bacterial tests were cut from the plates by using a computing numerical control (CNC) machine (Roland 3D Plotter MDX-50, Shizuoka, Japan).

### 3.3. Density and Mechanical Properties

The densities of the neat green LDPE matrix and the CPC were evaluated in accordance with ASTM D 792 using an analytical balance (SCALTEC, Göttingen, Germany) that contains an immersion vessel for the specimens in propanol. An average of six samples per condition were evaluated.

The mechanical properties of the biocomposites under a tensile load were performed using an Instron 5543 (High Wycombe, UK) Universal Mechanical Testing Equipment, according to the standard ASTM D 638. Prior to the tests, the samples were kept at room temperature for at least 48 h. The tests were conducted with a 1 kN load cell, a gauge length of 20 mm, and a crosshead speed of 5 mm/min. The elastic modulus was determined from the initial linear region of the stress/strain curve. Nine specimens per condition were evaluated and the results are presented in terms of mean ± standard deviation.

### 3.4. Thermal Properties

Differential scanning calorimetry (DSC) was used to determine the thermal properties of the biocomposite materials. The measurements were conducted in a TA Instruments DSC Q100 (New Castle, DE, USA) in order to analyze 3 to 6 mg of the sample. DSC analysis was performed under a flow of 50 mL/min of nitrogen atmosphere, and the heating rate used was 10 °C/min. Two heating ramps were tested; however, only the second one was analyzed for melting temperature (T_m_) and melting enthalpy (Δ*H_m_*).

The degree of crystallinity was calculated according to Equation (1). The melting enthalpy for 100% crystalline LDPE ($\Delta H_{m}^{0}$) is 293 J/g [58].

(1) $$\chi_{c}=\Delta H_{m}/\left(\Delta H_{m}^{0}\left(1-w\right)\right)\times 100$$

where Δ*H_m_* is the apparent fusion enthalpy for gram of biocomposite and w is the fraction of cork in the biocomposite.

### 3.5. Contact Angle Measurements

The water contact angles of the pristine bio-based LDPE and its biocomposite surfaces were determined through the sessile drop method using a goniometer (Data Physics OCA 15 Plus, Filderstadt, Germany). For each surface, at least 25 measurements were taken at (25 ± 2) °C with the drop size of ultra-pure water controlled at 4 µL [59].

### 3.6. Water Absorption

The water absorption (WA) was determined according to ASTM D 570 by weighing the specimens before and after immersion in ultra-pure water at room temperature for up to 14 days. The calculation of the water uptake was performed according to Equation (2).

(2) $$WA\;\left(\%\right)=\left[\left(m_{f}-m_{i}\right)/m_{i}\right]\times 100,$$

where $m_{i}$ is the mass in gram of the sample before the beginning of the assay and $m_{f}$ is the mass in gram of the sample after the test.

### 3.7. Antibacterial Activity

#### 3.7.1. Bacterial Strains and Culture Conditions

The selected bacteria were a model pathogen obtained from the Spanish Type Culture Collection (Valencia, Spain)—*S. aureus* CECT 976 and an industrial isolate from a fresh-cut salad process [60]—*P. putida*. *S. aureus* is a Gram-positive, round-shaped bacterium that is frequently associated with food poisoning, as well as hospital and community infections [61]. *P. putida* is a Gram-negative bacterium that is known to be responsible for chilled food spoilage [62,63]. 

Overnight cultures of *S. aureus* CECT 976 and *P. putida* were obtained via inoculation of 500 μL of a glycerol stock (kept at −80 °C) to a total volume of 200 mL of Tryptone Soy Broth (TSB; Merck KGaA, Darmstadt, Germany) and incubation at 30 °C with orbital agitation (AGITORB 200, Aralab, Rio de Mouro, Portugal). The cultures were then harvested via centrifugation (10 min, 3202× *g*) and the pellets were resuspended in fresh TSB. These bacterial suspensions were adjusted to an optical density (OD) of 0.01 at 600 nm (1:100 dilution from initial cell cultures at OD_600_ = 1) [64], which corresponds to about 5 × 10^6^ CFU mL^−1^.

#### 3.7.2. Surface Preparation

Round coupons (35 mm diameter) of pristine LDPE and its six biocomposites were rinsed in 70% (*v*/*v*) ethanol solution and twice in sterile water and left to dry in a laminar flow chamber for 24 h. The coupons were then placed in a desiccator until the moment of use to remove the remaining moisture. Control coupons were incubated in TSB at 30 °C for 3 days to confirm the sterility of the coupons.

#### 3.7.3. Biofilm Formation and Analysis

Disks of each produced material were placed into separate wells of a 6-well plate (VWR International, LLC, Carnaxide, Portugal) with the test surface facing up. To assess their antibacterial properties, 4 mL of the prepared suspension of *S. aureus* or *P. putida* was introduced into each well. Plates were incubated at 5 °C for 1 h to allow bacteria to attach to the surface materials [64]. After this step, the liquid was removed, the wells were refilled with 4 mL of TSB, and the plates were incubated for 3 days at 5 °C without agitation to allow biofilm development.

The biofilm spatial distribution and amount were determined by SEM and crystal violet (CV) staining, respectively. The spent medium was pipetted from the wells and the coupons were washed with 8.5 g/L NaCl to remove nonadherent cells.

The 3-day-old biofilms formed on pristine LDPE and CPC were analyzed using SEM (JEOL JSM-6010 LV, Tokyo, Japan). The biofilm samples were dehydrated with ethanol at different concentrations (10, 25, 40, 50, 70, 80, 90 and 100% (*v*/*v*)) [65], before observation. The coupons were then air-dried for 24 h in a desiccator and sputter-coated with platinum (Leica EM ACE600, Wien, Austria).

The amount of biofilm formed on the surfaces was quantitated using CV staining protocol adapted from previous works [23,66]. Briefly, biofilms formed on the surfaces were fixed with 1 mL of 100% ethanol (VWR International, LLC, Carnaxide, Portugal), which was removed after 15 min of contact. The wells were allowed to dry at room temperature, and 1 mL of 1% (*v*/*v*) CV (Merck KGaA, Darmstadt, Germany) solution was added per well and incubated for 5 min. The dye attached to the biofilm was solubilized in 1 mL of 33% (*v*/*v*) acetic acid (VWR International, LLC, Carnaxide, Portugal). Finally, 200 μL of each well was transferred to a 96-well plate (VWR International, LLC, Carnaxide, Portugal) and the absorbance at 570 nm was read in a microliter plate reader (SpectroStar Nano, BMG LABTECH, Madrid, Spain). When absorbance values exceeded 1, the samples were diluted in 33% (*v*/*v*) acetic acid, and the resulting measurements were corrected for the dilution factor and considered the absorbance value of the blank control (wells containing surfaces not exposed to bacteria). The biofilm amount on the biocomposites was expressed as a percentage in relation to pristine LDPE (control surface). Three independent experiments were performed with two replicates each.

### 3.8. Statistical Analysis

The analysis of the data was performed using the Shapiro-Wilk normality test. Since the results present a normal distribution for the density and mechanical tests, the contact angle measurements, and the antibacterial assays. One-way analysis of variance (ANOVA) and Tukey’s multiple comparisons test were used in Graphpad Prism 6.0 software.

## 4. Conclusions

The eco-friendly composites containing a bio-based LDPE and cork were produced via melt-based technologies and using different content of chitosan and coupling agent. This is the first study that addresses the potential of cork as an antibacterial agent and its synergetic effect using chitosan in polymer-matrix biocomposites. The results revealed a small decrease in the biocomposite density with the cork addition and an increase in the rate of water absorption (particularly in the presence of chitosan) due to the increase in the OH groups. The coupling agent PE-g-MA decreased the water absorption rate by reducing the free OH groups after the processing. At the surface level, a decrease in the hydrophobicity in the biocomposites was confirmed by the water contact angle that occurred due to the cork content. Chitosan slightly increased the contact angle of the cork-matrix system due to the decrease in surface roughness after processing. The mechanical properties of the cork biocomposites were improved by the use of chitosan, increasing the elastic modulus and the tensile strength without significantly affecting the tensile strain, suggesting its use in a wide range of applications. The cross-section morphology also revealed a good physical adhesion at the cork–matrix interface without voids or cracks and the fracture morphology, supporting the mechanical reinforcement provided by the chitosan and the coupling agent. The antibacterial activity results clearly indicate that *P. putida* and *S. aureus* biofilm formation were strongly reduced by using cork and cork/chitosan in the bio-based LDPE matrix. The development of new lightweight, eco-friendly, and sustainable biocomposite materials by using cork and chitosan to improve the bio-based LDPE behavior might be a promising solution for short-time food and cosmetic packaging and storage, healthcare, and in products that requires the reduction or absence of biofilm formation.

## Figures and Tables

**Table 1:** Melting temperatures and enthalpies, crystallization temperatures, and crystallinity degrees of CPCs with bio-based LDPE as the matrix.

	Crystallization		Second Heating		
	T_c_ (°C)	ΔH_c_ (J/g)	T_m_ (°C)	ΔH_m_ (J/g)	$\chi_{c}$
LDPE	100.3 ± 0.8	131.2 ± 4.3	104.4 ± 0.5	120.2 ± 3.2	41.0 ± 1.1
CPC5	100.4 ± 0.1	121.8 ± 2.3	104.8 ± 0.3	110.8 ± 3.0	39.8 ± 1.1
CPC20	100.8 ± 0.5	96.8 ± 5.4	104.8 ± 0.2	91.8 ± 5.8	39.1 ±2.5
CPC20CHT5	100.8 ± 0.1	98.2 ± 2.9	104.8 ± 0.2	88.2 ± 3.9	37.6 ± 1.7
CPC20CHT10	102.4 ± 1.9	98.0 ± 4.1	104.8 ± 0.03	91.5 ± 5.5	39.1 ± 2.3
CPC20CHT10MA2	108.3 ± 1.3	96.0 ± 1.7	99.1 ± 0.6	87.1 ± 4.1	37.2 ± 1.7
CPC20CHT10MA5	113.4 ± 0.1	104.7 ± 6.5	96.0 ± 0.1	94.3 ± 5.1	40.2 ± 2.2

**Table 2:** The composition and processing parameters of the CPC obtained through extrusion.

Composition	Constituents				Processing Conditions	
	LDPE (wt. %)	Cork (wt. %)	Chitosan (wt. %)	Coupling Agent (wt. %)	Temperature Profile (°C)	Screw Speed (rpm)
LDPE	100	0	0	0	100–130–135–140–145	50
CPC5	95	5	0	0		
CPC20	80	20	0	0		
CPC20CHT5	77.5	17.5	5	0		
CPC20CHT10	75	15	10	0		
CPC20CHT10MA2	74	14	10	2		
CPC20CHT10MA5	72.5	12.5	10	5		

## Data Availability

The data presented in this study are available on request from the corresponding author, upon reasonable request.

## Supplementary Materials

The following supporting information can be downloaded at: https://www.mdpi.com/article/10.3390/molecules28030990/s1, Table S1: Mechanical properties for the developed biocomposites in comparison with the bio-based LDPE matrix. The results are exhibit as mean ± standard deviation (SD).
