# Supplementary material for: Development of Cork Biocomposites Enriched with Chitosan Targeting Antibacterial and Antifouling Properties
Source: Molecules. 2023 Jan 18;28(3):990. doi: 10.3390/molecules28030990 (PMC9921838; doi:10.3390/molecules28030990)
Supplement: Supplementary file 1 [file molecules-28-00990-s001.zip › molecules-2140278-supplementary.pdf]

Supporting Information

# Development of Cork Biocomposites Enriched with Chitosan Targeting Antibacterial and Antifouling Properties

**Emanuel M. Fernandes [1,2,*], Flávia C. M. Lobo [1,2], Sara I. Faria [3,4], Luciana C. Gomes [3,4], Tiago H. Silva [1,2], Filipe J. M. Mergulhão [3,4] and Rui L. Reis [1,2]**

**Table S1** Mechanical properties for the developed biocomposites in comparison with the bio-based LDPE matrix. The results are exhibit as mean ± standard deviation (SD).

| Material | Tensile Modulus (MPa) | Max. Tensile Strength (MPa) | Strain at maximum load (%) |
|---|---|---|---|
| LDPE | 165.35 ± 19.55 | 12.06 ± 0.37 | 77.73 ± 5.93 |
| CPC5 | 175.84 ± 14.50 | 11.05 ± 0.65 | 18.76 ± 2.28 |
| CPC20 | 152.72 ± 13.62 | 7.65 ± 0.45 | 12.30 ± 0.86 |
| CPC20CHT5 | 185.62 ± 18.41 | 8.86 ± 0.15 | 11.43 ± 1.32 |
| CPC20CHT10 | 195.32 ± 12.88 | 8.72 ± 0.51 | 10.46 ± 0.60 |
| CPC20CHT10MA2 | 230.30 ± 0.15 | 11.99 ± 1.05 | 10.90 ± 0.64 |
| CPC20CHT10MA5 | 242.58 ± 16.64 | 12.75 ± 1.84 | 12.08 ± 1.84 |
